# 

**Published:** 1855-05

**Authors:** 


### RECEIPTS FROM MARCH 27 UP TO APRIL 27, 1855.

ILLINOIS

| | | | |
|---|---|---|---|
| Dr A W Rawson | $2 00 | Dr J Gregory | 2 00 |
| F W Hance | 2 00 | H Daniels | 2 00 |
| E Bennett | 6 00 | A Hull | 2 00 |
| E Smith | 2 00 | C Secrest | 2 00 |
| E K Boardman | 2 00 | R E Rica | 2 00 |
| W F Vermilion | 2 00 | N Lapp | 2 00 |
| G Coatsworth | 2 00 | | |

INDIANA

| | | | |
|---|---|---|---|
| Dr P Meyer | 2 00 | Dr J C Helm | 2 00 |
| Long & Ellis | 2 00 | J E Heald | 2 00 |
| R D Mauzy | 4 00 | A B Buchanan | 2 00 |

WISCONSIN

| | | | |
|---|---|---|---|
| Dr A Bodenstab | 2 00 | Dr S Spencer | 2 00 |
| N T Willard | 2 00 | R Morris | 2 00 |
| S A Pease | 2 00 | R Neilton | 2 00 |
| W Carren | 2 00 | | |

| | |
|---|---|
| Dr J Williamsen, Iowa, | $1 00 |
| E Van Foss, ,, | 2 00 |
| J D Blanchard, ,, | 2 00 |
| W Van Nuys, Kentucky | 2 00 |
| G B Dandson, Michigan | 1 00 |

